# Changes in the epidemiology of invasive fungal disease in a Pediatric Hematology and Oncology Unit: the relevance of breakthrough infections

**DOI:** 10.1186/s12879-023-08314-9

**Published:** 2023-05-25

**Authors:** Laura Calle-Miguel, Carmen Garrido-Colino, Begoña Santiago-García, Martha Patricia Moreno Santos, Henar Gonzalo Pascual, Beatriz Ponce Salas, Cristina Beléndez Bieler, Marisa Navarro Gómez, Jesús Guinea Ortega, Elena María Rincón-López

**Affiliations:** 1 Pediatric Infectious Diseases Section. Pediatrics Department. Hospital Materno, Infantil Gregorio Marañón. C/ O’, Donnell 48-50, 28009 Madrid, Spain; 2 Hospital General Universitario Gregorio Marañón (Pediatric Hematology and Oncology Unit. Pediatrics Department), Madrid, Spain; 3 Complutense University of Madrid, Madrid, Spain; 4 Hospital General Universitario Gregorio Marañón (Pediatric Infectious Diseases Unit. Pediatrics Department), CIBERINFEC, Instituto de Salud Carlos III, Madrid, Spain; 5 Hospital General Universitario Gregorio Marañón (Clinical Microbiology and Infectious Diseases Department), Madrid, Spain

**Keywords:** Invasive fungal disease, Children, Immunocompromised, Epidemiology

## Abstract

**Background:**

Invasive fungal disease (IFD) is a significant cause of morbimortality in children under chemotherapy or hematopoietic stem cell transplant (HSCT). The purpose of this study is to describe the changes in the IFD epidemiology that occurred in a Pediatric Hematology-Oncology Unit (PHOU) with an increasing activity over time.

**Methods:**

Retrospective revision of the medical records of children (from 6 months to 18 years old) diagnosed with IFD in the PHOU of a tertiary hospital in Madrid (Spain), between 2006 and 2019. IFD definitions were performed according to the EORTC revised criteria. Prevalence, epidemiological, diagnostic and therapeutic parameters were described. Comparative analyses were conducted using Chi-square, Mann-Whitney and Kruskal-Wallis tests, according to three time periods, the type of infection (yeast *vs* mold infections) and the outcome.

**Results:**

Twenty-eight episodes of IFD occurred in 27 out of 471 children at risk (50% males; median age of 9.8 years old, [IQR 4.9-15.1]), resulting in an overall global prevalence of 5.9%. Five episodes of candidemia and 23 bronchopulmonary mold diseases were registered. Six (21.4%), eight (28.6%) and 14 (50%) episodes met criteria for proven, probable and possible IFD, respectively. 71.4% of patients had a breakthrough infection, 28.6% required intensive care and 21.4% died during treatment.

Over time, bronchopulmonary mold infections and breakthrough IFD increased (*p*=0.002 and *p*=0.012, respectively), occurring in children with more IFD host factors (*p*=0.028) and high-risk underlying disorders (*p*=0.012). A 64% increase in the number of admissions in the PHOU (*p*<0.001) and a 277% increase in the number of HSCT (*p*=0.008) were not followed by rising rates of mortality or IFD/1000 admissions (*p*=0.674).

**Conclusions:**

In this study, we found that yeast infections decreased, while mold infections increased over time, being most of them breakthrough infections. These changes are probably related to the rising activity in our PHOU and an increase in the complexity of the baseline pathologies of patients. Fortunately, these facts were not followed by an increase in IFD prevalence or mortality rates.

**Supplementary Information:**

The online version contains supplementary material available at 10.1186/s12879-023-08314-9.

## Background

Invasive fungal disease (IFD) is one of the leading causes of morbidity and mortality among immunocompromised children. In recent years, there has been a significant increase in pediatric patients at risk, due to the extended use of immunosuppressive medications and a rising complexity of the baseline pathologies [1]. Children who receive chemotherapy for malignancy or undergo hematopoietic stem cell transplant (HSCT) are at the highest risk for an IFD, particularly invasive candidiasis and aspergillosis [1, 2]. In December 2019, the European Organization for Research and Treatment of Cancer and the Mycoses Study Group Education and Research Consortium (EORTC/MSGERC) published an update on the definitions of IFD, including specific considerations for pediatric patients [3]. Monitoring the local epidemiology is essential to understand the pediatric IFD burden to set up preventive measures, rationalize resources and implement institution-based infection control strategies [1, 4].

The management of IFD, especially mold infections, in immunocompromised children is a challenge. Signs and symptoms may be non-specific and often develop late during the disease progression [2]. Data on imaging tools for the diagnosis of IFD are scarce and the role of fungal biomarkers may differ from those used in adults [5–8]. Randomized clinical trials evaluating antifungal drugs rarely involve children; therefore, these drugs are often used "off-label" for compassionate use. Antifungal prophylaxis (AFP) has shown to improve outcomes of these patients, but, as well as for adults, breakthrough invasive fungal infections have emerged as a significant problem [9–11].

The Pediatric Hematology-Oncology Unit (PHOU) of Hospital General Universitario Gregorio Marañón (HGUGM) in Madrid (Spain) has experienced a rising activity and an increase in the complexity of patients’ pathologies in recent years. This study aims to describe IFD in children admitted to the PHOU, according to the latest EORTC criteria [3], analysing the breakthrough IFD rate and evaluating the changes over the study period. Secondary objectives were to compare yeast and mold infections and the characteristics of surviving and non-surviving patients.

## Methods

### Study design and population

Clinical data of all pediatric patients (from 6 months to 18 years old) diagnosed with IFD at the PHOU of the HGUGM from January 2006 to December 2019 were retrospectively reviewed. HGUGM is a tertiary hospital located in Madrid (Spain) with 120 pediatric beds and around 7000 pediatric inpatient admissions per year. The PHOU of HGUGM counts with 18 hospitalizations beds and has experienced a rising activity in recent years, due, partially, to the creation of an adolescent unit and the accreditation as a National Reference Center for hereditary erythropathologies. The number of admissions per year in the PHOU has progressively increased from 343 in 2006 to 853 in 2019.

The information collected from the patients included: demographics, underlying condition, IFD host factors, AFP, laboratory, microbiological and radiological findings, antifungal therapy and clinical outcome. Deaths observed during the IFD treatment period were analyzed.

### Definitions

Cases were defined as proven, probable and possible IFD based on the last update in 2019 of the consensus definitions of IFD from the EORTC/MSGERC. Proven disease required histopathologic or microbiologic documentation of infection from tissue obtained by biopsy or autopsy, or an isolation from a culture sample obtained from a normally sterile site. Probable disease was defined as the presence of host factors, clinical features and mycological evidence of an IFD. Possible disease required proper host factors and sufficient clinical evidence compatible with IFD [3].

Host factors for IFD were those defined by the EORTC/MSGERC consensus: a recent history of prolonged neutropenia (<500 neutrophils/µL for >10 days), hematologic malignancy, allogeneic HSCT or solid organ transplant, prolonged use of corticosteroids (≥0.3 mg/kg for ≥ three weeks in the past 60 days), treatment with B-cell o T-cell immunosuppressants, severe hereditary immunodeficiency and graft-versus-host disease (GVHD) [3].

The risk for IFD represented the probability of IFD for each underlying condition. Patients considered at high-risk (≥10%) for IFD were those with acute myeloid leukemia (AML), high-risk or relapsed or acute lymphoblastic leukemia (ALL), severe aplastic anemia, myelodysplastic syndrome or those who underwent an allogeneic HSCT or developed a GVHD. Patients with non-Hodgkin lymphoma, standard-risk ALL or autologous HSCT were considered at low-risk (≤5%) for IFD. In opposition, patients with solid organ tumors (SOT), like brain tumors or Hodgkin lymphoma, were classified as sporadic risk [12, 13].

The global prevalence of IFD was calculated dividing the number of IFD cases by the total number of patients at risk. Moreover, the ratio of IFD cases per 1000 admissions in the PHOU was calculated globally and by time periods. The activity in the PHOU was measured using the number of admissions and HSCT carried out among periods.

Breakthrough IFD was defined as any IFD occurring during exposure to an antifungal drug, according to the recent definitions by the MSGERC [11]. In our institution protocol, used along the study period, oral or intravenous posaconazole, with therapeutic drug monitoring, and intravenous micafungin are considered the first choices for AFP in patients at high-risk for IFD. Liposomal amphotericin B is usually used for empirical or pre-emptive treatment in the setting of persistent neutropenic fever over 96 hours of significant respiratory symptoms, and voriconazole is the treatment of choice if an invasive aspergillosis is proven or probable.

### Ethics

This study was conducted in accordance with the 1964 Declaration of Helsinki and its subsequent amendments. Ethics approval was obtained from the Clinical Research Ethics Committee at HGUGM (date 5^th^ October 2020/ Number IFI-HOI-2020).

### Statistical analyses

Descriptive analyses were performed using frequencies and proportions for categorical variables and medians and interquartile ranges (IQR) for continuous variables. Comparative analyses were conducted according to time periods (three 56-months periods: January 2006 to August 2010 *vs* September 2010 to April 2015 *vs* May 2015 to December 2019), the type of infection (yeast *vs* mold infections), and outcome (survivors *vs* non-survivors). Categorical variables were compared using the chi-square or the Fisher exact test, as appropriate. Continuous dicotomic variables were evaluated with the Mann-Whitney U test. Kruskal-Wallis test, followed by *posthoc* analyses, was used when comparing variables among the three time periods. Statistical analyses were performed using IBM SPSS Statistics software (Statistical Package for the Social Sciences) version 23.0. The statistical significance level was defined as a two-tailed *p-*value <0.05.

## Results

From January 2006 to December 2019, a total of 471 children at risk for an IFD were followed and 90 HSCT (65 allogeneic, 25 autologous) were carried out at the PHOU. The underlying condition of these children at risk were: 26 AML, 66 ALL, 333 SOT and 46 HSCT performed in non-malignant diseases. Twenty-six children had one episode of IFD and one child had two episodes separated by four years, corresponding to 28 episodes overall. The median age at diagnosis was 9.8 years (IQR 4.9-15.1 years) and 50% were males. The global prevalence of IFD was 5.9%, and differed according to the underlying condition: 23.1% (6/26) for AML, 12.1% (8/66) for ALL and 1.8% (6/333) for SOT. The prevalence in those who required a HSCT was 13.3% (12/90), being 16.9% (11/65) for allogeneic and 4% (1/25) for autologous HSCT. Twenty-three were mold and five were yeast infections, with a prevalence of 4.9% and 1.1%, respectively.

There was a global ratio of 4.1 cases of IFD per 1000 admissions in the PHOU and decreased by 25% (*p*=0.674) between the first and the last study periods (Table 1). In contrast, there was a significant rising activity in the PHOU, with a 64% increase in the number of admissions (*p*<0.001) and 277% in the number of HSCT carried out (*p*=0.008). In the last period, children had more IFD host factors (*p*=0.028) and high-risk underlying disorders (*p*=0.012), being the cases of breakthrough IFD more frequent (*p*=0.012). All yeast infections took place during the first period (55.6% of cases in period 1), whereas in the second and third periods all IFD episodes were mold infections (*p*=0.002).

**Table 1:** Period analysis of the clinical activity at the PHOU and IFD epidemiological and clinical characteristics

**PHOU activity**	Period 1 (1/2006 - 8/2010)	Period 2 (9/2010 – 4/2015)	Period 3 (5/2015- 12/2019)	*p*
No. admissions	1881	2041	3077	**<0.001**
No. HSCT `	13	28	49	**0.008**
No. IFD /1000 admissions	4.8	3.9	3.6	0.674
**IFD characteristics**	Period 1 *n=*9	Period 2 *n=*8	Period 3 *n=*11	*p*
**Type of infection**				
Mold infection	4 (44.4%)	8 (100%)	11 (100%)	**0.002**^(1)^
Yeast infection	5 (55.6%)	0 (0%)	0 (0%)	**0.002**^(1)^
**Site of disease**				
Bloodstream	5 (55.6%)			**0.002**^(1)^
Bronchopulmonary	4 (44.4%)	8 (100%)	11 (100%)	**0.002**^(1)^
Rhino-sinusitis			2 (18.2%)	0.201
**Age (years)**	6.5 (1.4-14.5)	8.9 (3.2 -11.2)	12.3 (9.8-15.7)	0.173
**Number of host factors**	1 (0-4)	4 (1-5)	3 (1-5)	**0.028**^(2)^
**Host factors**				
Prolonged neutropenia	1 (20%)	5 (71.4%)	3 (27.3%)	0.182
Hematologic malignancy	2 (22.2%)	4 (50%)	8 (72.7%)	0.077
Allogeneic HSCT	2 (22.2%)	5 (62.5%)	4 (36.4%)	0.264
Prolonged use of steroids	1 (33.3%)	3 (37.5%)	7 (63.6%)	0.505
Acute GVHD	0 (0%)	3 (37.5%)	2 (18.2%)	0.178
Chronic GVHD	1 (11.1%)	1 (12.5%)	2 (18.2%)	1.000
**Risk stratification**				
High-risk	3 (33.3%)	7 (87.5%)	10 (90.9%)	**0.012**^(3)^
Low-risk	2 (22.2%)	1 (12.5%)	0 (0%)	0.258
Sporadic	4 (44.4%)	0 (0%)	1 (9.1%)	0.045^(2)^
**Breakthrough IFD**	3 (33.3%)	7 (87.5%)	10 (90.9%)	**0.012**^(3)^
**IFD diagnosis**				
Proven IFD	5 (55.6%)	1 (12.5%)	0 (0%)	**0.007**^(1)^
Probable IFD	1 (11.1%)	4 (50%)	3 (27.3%)	0.328
Possible IFD	3 (33.3%)	3 (37.5%)	8 (72.7%)	0.060
**Treatment duration (days)**	51 (16-70)	23 (8-43)	67 (42-72)	**0.037**^(4)^
**Outcome**				
PICU admission	1 (11.1%)	5 (62.5%)	2 (18.2%)	0.066
Mechanical ventilation	0 (0%)	3 (37.5%)	1 (9.1%)	0.080
Inotropic support	0 (0%)	2 (25%)	1 (9.1%)	0.352
*Exitus*	0 (0%)	4 (50%)	2 (18.2%)	0.047^(5)^

Data presented correspond to n (%) for categorical parameters and median (IQR) for continuous variables. Bold numbers: values that are statistically significant

*Abbreviations*: *GVHD* Graft-versus-host disease, *HSCT* Hematopoietic stem cell transplant, *IFD* Invasive fungal disease, *IQR* Interquartile range, *PICU* Pediatric Intensive Care Unit, *PHOU* Pediatric Hematology-Oncology Unit

^(^^1)^difference between group 1 and group 3 in the *post-hoc* analyses (*p*=0.008)

^(2)^group 1 is different from groups 2 and 3 in the *post-hoc* analyses

^(^^3)^difference between group 1 and group 3 in the *post-hoc* analyses (*p*=0.017)

^(4)^difference between group 2 and group 3 in the *post-hoc* analyses (*p*=0.019)

^(5)^no differences between groups in the *post-hoc* analyses

Most episodes (71.4%) occurred in children with underlying conditions considered at high-risk for IFD. Fourteen (50%) corresponded to children with blood malignancies: ALL *n=*8 (standard-risk ALL *n=*2; relapsed ALL *n=*3; high-risk ALL *n=*3) and AML *n=*6. Eight (28.6%) occurred in children with non-malignant blood disorders, all of them after allogeneic HSCT (sickle cell disease *n=*4, aplastic anemia *n=*2, Fanconi anemia *n=*1, β-thalassemia major *n=*1), and six (21.4%) were children with SOT (neuroblastoma *n=*3, osteosarcoma *n=*2, sarcoma *n=*1). One of the IFD episodes in patients diagnosed with ALL occurred during the induction treatment (standard-risk ALL), and the 3 cases in high-risk ALL occurred after a HSCT. The most common host factors for IFD were blood malignancies (50%), prolonged use of corticosteroids (50%), allogeneic HSCT (39.3%; median time between HSCT and IFD 43 days [IQR 14 - 253 days]) and prolonged neutropenia (39.1%); median time of neutropenia 16 days [IQR 12.5 - 23 day]). Eight patients (28.6%) were not neutropenic at the time they developed the IFD. Other host factors were: treatment with T-cell immunosuppressants (28.6%), acute GVHD (17.9%) and chronic GVHD (14.3%). More than a third of the patients (39.3%) had three or four IFD host factors and 7.1% had five or more.

Twenty children (71.4%) were receiving AFP, including 19/20 high-risk children and 1/3 low-risk children. The characteristics of these 20 breakthrough IFD episodes are described in Table 2. Levels of posaconazole were subtherapeutic in both patients at the time they developed the IFD. The global prevalence of breakthrough IFD was 4.2%, and according to the underlying condition, it was 23.1%, 9.1%, 0.3% and 12.2% for AML, ALL, SOT and HSCT groups, respectively. All the breakthrough episodes were mold infections. Forty percent of these episodes were defined as probable and 60% as possible disease.

**Table 2:** Characteristics of breakthrough IFD episodes (*n=*20)

**Epidemiological characteristics**	
Male sex	10 (50%)
Age (years)	9.5 (6.5-15)
**Underlying conditions**	
ALL	6 (30%)
AML	6 (30%)
SOT	1 (5%)
Non-malignant blood disorders	7 (35%)
**HSCT**	11 (55%)
Allogeneic	10/11
Autologous	1/11
**Risk stratification**	
High-risk	19 (95%)
Low-risk	1 (5%)
Sporadic	0
**Type of AFP**	
Micafungin (intravenous formulation)	13 (65%)
Fluconazole (oral formulation)	4 (20%)
Posaconazole (oral formulation)	2 (10%)
Itraconazole (oral formulation)	1 (5%)
**IFD diagnosis**	
Proven IFD	0
Probable IFD	8 (40%)
Possible IFD	12 (60%)
**Type of infection**	
Mold infection	20 (100%)
Yeast infection	0
**Site of disease**	
Bronchopulmonary	20 (100%)
Rhino-sinusitis	2 (10%)
**Outcome**	
PICU admission	6 (30%)
Mechanical ventilation	3 (15%)
Inotropic support	2 (10%)
*Exitus*	5 (25%)

Data presented correspond to n (%) for categorical parameters and median (IQR) for continuous variables

*AFP* Antifungal prophylaxis, *ALL* Acute lymphoblastic leukemia, *AML* Acute myeloid leukemia, *HSCT* Hematopoietic stem cell transplant, *IFD* Invasive fungal disease, *IQR* Interquartile range, *PICU* Pediatric Intensive Care Unit, *SOT* Solid organ tumor

In 11 episodes (39.3%), a clinically relevant pathogen was identified by culture or polymerase chain reaction (PCR): five culture-positive yeast infections (*Candida albicans n=*2; *C. parapsilosis n=*2; *C. kefyr n=*1), one culture-positive mold infection (*Aspergillus ustus*) and five PCR-positive mold infections (*Aspergillus* spp *n=*3; *Cunninghamella* spp *n=*1; *Myriangiales* spp *n=*1). According to EORTC criteria, the final diagnosis was proven IFD in six episodes (21.4%; prevalence 0.2%), probable in eight (28.6%; prevalence 1.7%), and possible in 14 (50%; prevalence 3%).

Table 3 shows the differences between yeast and mold infections. All yeast infections were candidemia (*n=*5; 17.9%), whereas all mold infections caused bronchopulmonary disease (*n=*23; 82.1%), being associated with rhino-sinusitis in two cases. Children with mold infections had a higher number of IFD host factors (median 3 *vs* 1; *p*=0.001) and had more frequently underlying high-risk disorders (87% *vs* 0%; *p*=0.001). All yeast and only one mold infection met the criteria for proven IFD (100% vs 4.3%, *p*=0.001), the last one due to the findings in a necropsy compatible with an invasive aspergillosis. There was a non-significant trend towards higher mortality in mold than yeast infections (26.1% *vs* 0%, *p=*0.553). Table 4 shows the radiological and microbiological findings of mold IFD.

**Table 3:** Differences between yeast and mold infections

	Yeast infections (*n=*5)	Mold infections (*n=*23)	***p***
**Site of disease**			
Bloodstream disease	5 (100%)	0 (0%)	**<0.001**
Bronchopulmonary disease	0 (0%)	23 (100%)	**<0.001**
Sinonasal disease	0 (0%)	2 (8.7%)	1.000
**Age (years)**	1.8 (0.8-10.9)	10.5 (6.6-15.2)	0.053
**Number of host factors**	1 (0-1)	3 (2-4)	**0.001**
**Host factors**			
Prolonged neutropenia	0 (0%)	9 (42.9%)	0.502
Hematologic malignancy	1 (20%)	13 (56.5%)	0.326
Allogeneic HSCT	0 (0%)	11 (47.8%)	0.125
Prolonged use of corticosteroids	No data	11 (50%)	-
Acute GVHD	0 (0%)	5 (21.7%)	0.550
Chronic GVHD	0 (0%)	4 (17.4%)	1.000
**Risk stratification**			
High-risk	0 (0%)	20 (87%)	**0.001**
Low-risk	1 (20%)	2 (8.7%)	0.459
Sporadic	4 (80%)	1 (4.3%)	**0.001**
**Breakthrough IFD**	0 (0%)	20 (87%)	**0.001**
**Laboratory parameters**			
Hemoglobin (g/dl)	9.5 (8.1-11)	8.9 (7.9-10.7)	0.880
Platelets x10^3^/µL	94 (71.5-163.5)	39.5 (19.8-66.5)	**0.016**
Leukocytes/µL	1375 (1050-5350)	1200 (200-5950)	0.650
Neutrophils/µL	440 (40-3250)	100 (0-4250)	0.705
Lymphocytes/µL	860 (223-1530)	500 (100-1050)	0.496
CRP (mg/dl) at diagnosis	1.1 (0.2-6.3)	6.7 (2.6-12.7)	0.113
Maximum CRP (mg/dl)	13.9 (4-13.9)	12 (3.3-20.7)	0.787
Procalcitonin (ng/ml) at diagnosis	2.2 (2.2-2.2)	0.12 (0.1-0.2)	0.333
Maximum procalcitonin (ng/ml)	2.2 (2.2-2.2)	0.6 (0.3-1.1)	0.400
**IFD diagnosis**			
Proven IFD	5 (100%)	1 (4.3%)	**0.001**
Probable IFD	0 (0%)	8 (34.8%)	0.281
Possible IFD	0 (0%)	14 (60.9%)	**0.041**
**Treatment duration**	32 (15.5-60.5)	43.5 (25-71)	0.488
**Outcome**			
PICU admission	1(20%)	7 (30.4%)	1.00
Mechanical ventilation	0 (0%)	4 (17.4%)	1.00
Inotropic support	0 (0%)	3 (13%)	1.00
*Exitus*	0 (0%)	6 (26.1%)	0.553

Data presented correspond to n (%) for categorical parameters and median (IQR) for continuous variables. Bold numbers: values that are statistically significant

*Abbreviations*: *CRP* C-reactive protein, *GVHD* Graft-versus-host disease, *HSCT* Hematopoietic stem cell transplant, *IFD* Invasive fungal disease, *IQR* Interquartile range, *PICU* Pediatric Intensive Care Unit

**Table 4:** Diagnostic tools used in mold infections

Case	Year	IFD diagnosis	GM in serum	GM in BAL	BDG^b^ (pg/ml)	Mycological data: pathogen, technique, sample (number)	Radiological findings. Unilateral or bilateral pattern
1	2006	Possible	0.25	ND	NA		Bilateral. Nodules
2	2007	Possible	0.39	ND	NA		Unilateral. Alveolar consolidation
3	2007	**Probable**	3.30^c^	ND	NA		Bilateral. Pulmonary infiltrates, pseudonodules
4	2007	Possible	0.51	ND	NA		Bilateral. Nodules
5	2011	**Probable**	4.34^c^	ND	NA		Bilateral. Bronchoceles
6	2011	**Probable**	1.48^c^	ND	NA		Bilateral. Pulmonary infiltrates, pseudonodules
7^a^	2012	**Probable**	2.20^c^	ND	NA	*Cunninghamella* spp, PCR, pleural effusion (2)	Unilateral. Alveolar consolidation
8^a^	2013	Possible	0.42	ND	NA		Bilateral. Alveolar consolidations, pulmonary infiltrates, pseudonodules
9	2014	Possible	0.48	0.23	NA	*Aspergillus* spp, PCR, BAL (1)	Unilateral. Pulmonary infiltrates
10^a^	2014	**Proven**	ND	ND	NA		Bilateral. Alveolar consolidations, cavity
11^a^	2015	Possible	0.25	0.19	NA		Bilateral. Pseudonodules, halo sign
12	2015	**Probable**	0.69	2.24^c^	NA		Unilateral. Alveolar consolidation
13	2015	Possible	0.30	0.10	NA	*Aspergillus* spp*,* PCR, BAL (1)	Bilateral. Pulmonary infiltrates, nodules, halo sign
14^a^	2016	Possible	0.60	0.21	NA		Bilateral. Alveolar consolidations, pulmonary infiltrates
15	2016	**Probable**	0.10	1.84^c^	NA		Unilateral. Alveolar consolidation, pulmonary infiltrates
16^a^	2016	**Probable**	7.22^c^	ND	NA	*Aspergillus ustus*, culture, BAS (1)	Bilateral. Pulmonary infiltrates, nodules
17	2016	**Probable**	0.24	2.17^c^	NA		Bilateral. Pulmonary infiltrates, pseudonodules
18	2017	Possible	0.11	0.09	NA	*Myriangiales* spp, PCR, BAL (1)	Bilateral. Pulmonary infiltrates, nodules, pseudonodules, cavities
19	2017	Possible	0.09	0.15	NA	*Aspergillus* spp, PCR, BAL (1)	Unilateral. Nodules, halo sign
20	2017	Possible	0.09	0.16	NA		Unilateral. Alveolar consolidation, pulmonary infiltrates
21	2018	Possible	0.16	0.13	3.30		Unilateral. Alveolar consolidation
22	2019	Possible	0.21	0.19	90.50^c^		Unilateral. Pulmonary infiltrates
23	2019	Possible	0.15	0.28	62.10^c^		Unilateral. Alveolar consolidation, nodules, halo sign

*Abbreviations*: *BAL* Bronchoalveolar lavage, *BAS* Bronchial aspirate, *BDG* (1-3)-β-D-glucan, *GM* Galactomannan, *NA* Not available, *ND* Not done, *PCR* Polymerase chain reaction

^a^non-survival cases

^b^determined by Wako® manufacturer (positive cut off > 11.1 pg/ml)

^c^positive results. In bold font: proven and probable cases

Liposomal amphotericin B and voriconazole were the first options for IFD treatment in 85.7% (*n=*24) and 25% (*n=*7) of cases, respectively, being used in combination in 14.2% of cases. Two-thirds of the children experienced a change of treatment (67.9%), mostly based on voriconazole (68.4%), and 10.7% needed a third-line option. The median duration of treatment was 43 days (IQR 19.5-69 days).

Regarding outcomes, 8 patients (28.6%) required admission to the Pediatric Intensive Care Unit (PICU), 4 (14.3%) needed mechanical ventilation and 3 (10.7%), inotropic support. One patient required pleural effusion drainage but no cases required surgery. Six patients (21.4%) died during the IFD episode, all of which had mold infections, although death was directly attributed to the IFD in only one of them. Other causes of mortality were: disease progression (*n=*2), refractory GVHD (*n=*2) and a bacterial sepsis with multi-organic failure (*n=*1). Table 5 compares the characteristics between surviving and non-surviving children.

**Table 5:** Differences between surviving and non-surviving patients

	Survivors (*n=*22)	Non-survivors (*n=*6)	*p*
**Type of infection**			
Mold infection	17 (77.3%)	6 (100%)	0.553
Yeast infection	5 (22.7%)	0 (0%)	
**Site of disease**			
Bronchopulmonary disease	17 (77.3%)	6 (100%)	0.553
Bloodstream disease	5 (22.7%)	0 (0%)	
**Epidemiological data**			
Age (years)	10.1 (6.3-14.9)	6.9 (2.2-16)	0.566
Sex: male	10 (45.5%)	4 (66.7%)	0.648
**Number of host factors**	2 (1-3.3)	3 (2.5-4)	0.188
**Host factors**			
Prolonged neutropenia	7 (38.9%)	2 (40%)	1.000
Hematologic malignancy	9 (40.9%)	5 (83.3%)	0.165
Allogeneic HSCT	8 (36.4%)	3 (50%)	0.653
Prolonged use of corticosteroids	7 (43.8%)	4 (66.7%)	0.635
Acute GVHD	3 (13.6%)	2 (33.3%)	0.285
Chronic GVHD	2 (9.1%)	2 (33.3%)	0.191
**Risk stratification**			
High-risk	15 (68.2%)	5 (83.3%)	0.640
Low-risk	2 (9.1%)	1 (16.7%)	0.530
Sporadic	5 (27.7%)	0 (0%)	0.553
**Antifungal prophylaxis**	15 (68.2%)	5 (83.3%)	0.640
**Laboratory parameters**			
Haemoglobin (g/dl)	8.9 (8.2-11.2)	8.2 (7.5-10.5)	0.243
Platelets x10^3^/µL	51 (27.5-114)	40.5 (10.3-66.5)	0.221
Leukocytes/µL	1200 (250-1200)	1350 (175-5000)	0.748
Neutrophils/µL; median (IQR)	200 (7-3975)	100 (0-4250)	0.516
Lymphocytes/µL; median (IQR)	500 (200-1320)	450 (100-950)	0.823
CRP (mg/dl) at diagnosis	3.9 (0.7-8.6)	10.1 (4.8-33.3)	0.056
Maximum CRP (mg/dl)	11.5 (2.2-20.1)	13.5 (10.1-34.7)	0.238
Procalcitonine (ng/ml) at diagnosis	0.1 (0.07-0.7)	0.2 (0.09-4.2)	0.688
Maximum procalcitonine (ng/ml)	0.7 (0.3-1.8)	0.6 (0.3-4.2)	0.556
GM in serum (mold infections)	0.25 (0.13-0.6)	0.6 (0.34-4.7)	0.085
GM in BAL (mold infections)	0.19 (0.13-1.84)	0.2 (0.19-0.2)	0.923
**IFD diagnosis**			
Proven IFD	5 (22.7%)	1 (16.7%)	1.000
Probable IFD	6 (27.3%)	2 (33.3%)	1.000
Possible IFD	11 (50%)	3 (50%)	1.000
**Treatment duration (days)**	51 (37-72)	11 (6-32)	**0.003**
**Outcome**			
PICU admission	4 (18.2%)	4 (66.7%)	**0.038**
Mechanical ventilation	0 (0%)	3 (50%)	**0.006**
Inotropic support	1 (4.5%)	3 (50%)	**0.022**

Data presented correspond to n (%) for categorical parameters and median (IQR) for continuous variables. Bold numbers: values that are statistically significant

*Abbreviations*: *CRP* C-reactive protein, *GM* Galactomannan, *GVHD* Graft-versus-host disease, *HSCT* Hematopoietic stem cell transplant, *IFD* Invasive fungal disease, *IQR* Interquartile range, *PICU* Pediatric Intensive Care Unit

## Discussion

The present study describes 28 episodes of IFD in 27 children out of 471 patients at risk in a PHOU during 14 years. Data about global prevalence, the breakthrough IFD rate, clinical characteristics, diagnosis and treatment of IFD in these children receiving chemotherapy or undergoing HSCT were described. Yeast infections decreased over time, whereas mold infections increased, being breakthrough IFD the majority of them, occurring in children with high-risk pathologies and numerous host factors. A rising activity in the PHOU and an increasing complexity of patients’ pathologies over the study period were not followed by an increase in the rate of IFD cases per 1000 admissions in the PHOU or in the mortality rates.

The global prevalence of IFD was 5.9%, being higher in children with AML (23.1%) and in those who underwent allogeneic HSCT (16.9%). The global prevalence of IFD in children with cancer and HSCT recipients ranges from 3.4-7.2% [1, 10, 14]. The wide variety of immune dysfunctions related to underlying conditions, institutional variations in diagnostic and supportive care practices and the inconsistencies in diagnostic criteria make the estimation of this prevalence very challenging [1, 3, 14–19]. Our study´s prevalence in different groups of patients was similar to that reported recently by Bartlett *et al* [16]. Other studies have also shown that AML, allogeneic HSCT and high-risk ALL have an exceptionally high risk for IFD [2, 10, 13, 19–22].

Several patients in our study, especially those with mold infections, had numerous host factors for developing an IFD. It is known that IFD in children rarely occurs in the presence of an isolated host factor [2]. Notably, we detected 20 episodes of breakthrough IFD, with an overall rate of 4.2%. All of them were bronchopulmonary mold infections and 80% of these patients were receiving a mold-active agent. Breakthrough IFD is an emerging significant problem in patients who receive systemic antifungals, ranging from 1.6 to 7.7% for proven and possible breakthrough infections [9, 23–27]. Nevertheless, the validity of fungal biomarkers may differ in such cases and rates can increase up to 13% when including possible infections [9, 28]. Invasive mold infection are the most common breakthrough IFD [23, 24, 26]. Primary AFP is mainly based on posaconazole, but the concern about pharmacological interactions and toxicity of triazoles has led to a search for alternatives [12, 13, 29]. Liposomal amphotericin and micafungin have been shown to be safe and effective options [12, 26, 27].

Prevalence of yeast and mold infections was 1.1% and 4.9%, respectively. We identified five episodes of proven bloodstream candidemia; all of them took place during the first study period in children with a central venous catheter, and 60% were non-*C. albicans* species. These findings are consistent with recent reports, which describe a decrease in yeast infections in the past decade, attributed to the extended use of AFP, improved environmental strategies and infection control measures during line emplacement. This fact, together with the increasing number of children under immunosuppressive medications or HSCT, have led mold infections to replace invasive candidiasis as the most frequent IFD [10, 15, 16, 18, 20, 29, 30]. The predominance of non-*C. albicans* in children has been attributed to the affinity of *C. parapsilosis* for central venous catheter [2, 14, 16, 29].

Twenty-three episodes were bronchopulmonary mold infections and two were associated with rhino-sinusitis. Most of them were possible or probable infections. The diagnosis of mold IFD in children is a challenge, as only 30-50% of cases meet the criteria for a proven or probable disease [14, 16, 19, 21]. In our study, pulmonary infiltrates and consolidations were the most frequent radiological signs whereas the halo sign and cavities appeared in 17.4% and 8.7% of cases. Other studies have shown that radiological findings in pediatric IFD are unspecific and adult hallmarks are less common, with the halo sign appearing in less than 15% of images and the air crescent sign being very rarely observed [7, 13–15, 18, 20].

In our study, only one child met the criteria for proven mold disease (based on necropsy findings), 8 were classified as probable disease (based on seric galactomannan [GM; 5/8 cases] or GM in bronchoalveolar lavage [BAL; 3/8]) and 14 as possible cases (including four children with a single positive PCR in BAL and two with positive serum (1-3)-β-D-glucan [BDG]). Reaching a proven mold disease diagnosis is generally unfeasible to pediatric patients [3]. BAL has positioned itself as a safe technique, useful for culture, GM and PCR testing [31, 32], but the detection of *Aspergillus* by PCR requires several positive tests to support the diagnosis of probable IFD [3]. The threshold of the promising BDG may vary according to the age, etiology, specimens and manufacturers, and it is not currently recommended to provide evidence of an invasive mold disease in pediatric patients [3, 8, 13, 29, 33]. The exposure to mold-active AFP reduces the sensitivity of GM assay and it may be a reason for the high proportion of possible IFD [3, 8, 13, 20, 31].

Antifungal agents and treatment duration in our study followed pediatric guidelines [13, 20, 31, 34–38]. The high variability in days of therapy may be explained because the length of treatment is not well defined in mold infections [20]. The prompt initiation of empiric antifungal therapy is critical in a suspected IFD to reduce mortality, being a pre-emptive treatment is a safe strategy to avoid overuse of antifungals [10, 13, 20, 39]. In our study, six patients (21.4%) died during antifungal treatment, all diagnosed with mold infections, one of these cases was directly related to the IFD. Despite the rising activity in the PHOU at HGUGM and an increase in the complexity of pathologies and in the breakthrough IFD rates, mortality rates did not increased. Mortality in the second period was higher but no differences in the comparison between groups were found. The overall case-fatality rate attributable to IFD is very variable, reaching 10-25% in yeast infections and 20-50% in mold infections [1, 10, 16, 19, 20]. The patients who died without completing treatment can explain the shorter duration of treatment in period 2.

This study has several limitations. First of all, it is a retrospective study and data are limited to the information available in medical records. Secondly, it is a single-center study with a relatively small sample size; however, these data are probably representative of the characteristics of IFD in pediatric oncohematologic patients, considering that it was done in a tertiary hospital during a long study period. The activity of the PHOU was analysed by non-specific measurements, as the total number of admissions and the HSCT performed. In addition, the incidence of IFD in each risk category was not calculated considering that the risk of each patient can change during the treatment, but prevalence was estimated according to the underlying condition. Finally, a multidisciplinary management between experts in pediatric oncologic and infectious diseases, that maintain a high level of suspicion of IFD in children with numerous host factors, could have led to an over-diagnosis of the cases of possible IFD, taking into account the low specificity of radiological findings in children. However, a prompt treatment of IFD suspected cases is justified given the severity and the mortality rates of this entity and can explain the stable mortality rates within the study period.

In conclusion, this study offers a good picture of IFD in children receiving chemotherapy or undergoing HSCT. We observed a decrease in yeast infections during the study time with an increase in the proportion of mold infections. The rising activity and complexity in our PHOU led to an increase in the breakthrough IFD rates, but not in the number of IFD/1000 admissions in the PHOU or in the mortality rates. Local epidemiology knowledge of IFD is essential to implement appropriate therapeutic interventions early and improve survival in these children.

## Supplementary Information

**Additional file 1.**

## Data Availability

The datasets used and/or analysed during the current study are available from the corresponding author on reasonable request.

## Abbreviations

AFP: antifungal prophylaxis
ALL: acute lymphoblastic leukemia
AML: acute myeloid leukemia
BAL: bronchoalveolar lavage
BDG: (1-3)-β-D-glucan
CRP: C-reactive protein
EORTC: European Organization for Research and Treatment of Cancer
GM: galactomannan
GVHD: graft-versus-host disease
HSCT: hematopoietic stem cell transplant
HGUGM: hospital General Universitario Gregorio Marañón
IFD: invasive fungal disease
IQR: interquartile range
MSGERC: Mycoses Study Group Education and Research Consortium
PCR: polymerase chain reaction
PHOU: pediatric Hematology-Oncology Unit
PICU: pediatric Intensive Care Unit
SOT: solid organ tumor
