# Supplementary material for: Changes in the epidemiology of invasive fungal disease in a Pediatric Hematology and Oncology Unit: the relevance of breakthrough infections
Source: BMC Infect Dis. 2023 May 25;23:348. doi: 10.1186/s12879-023-08314-9 (PMC10210274; doi:10.1186/s12879-023-08314-9)
Supplement: Supplementary file 1 — Additional file 1. [file 12879_2023_8314_MOESM1_ESM.docx]

Pediatric IFD Study Group:

- Teresa Hernández-Sampelayo Matos^ac^
- María del Mar Santos Sebastián^a^
- Jesús Saavedra Lozano^ac^
- David Aguilera Alonso^a^
- Elena Cela de Julián^bc^
- Cristina Mata Fernández^b^
- Marina García Morín^b^
- Jorge Huerta Aragonés^b^
- Eduardo Bardón Cancho^b^.

Affiliations

1. Hospital General Universitario Gregorio Marañón (Pediatric Infectious Diseases Unit. Pediatrics Department), Madrid, Spain. CIBERINFEC, Instituto de Salud Carlos III.
2. Hospital General Universitario Gregorio Marañón (Pediatric Hematology and Oncology Unit. Pediatrics Department), Madrid, Spain.
3. Complutense University of Madrid, Spain.
